# Oxaliplatin-induced neuropathic pain in cancer: animal models and related research progress

**DOI:** 10.3389/fphar.2025.1609791

**Published:** 2025-05-30

**Authors:** Yuxin Jiang, Jie Shi, Wenping Wang, Haozhe Piao, Huini Yao, Jun Yu, Zhenzhu Zhai, Qian Liu, Ningxin Li, Jiaqing Fu, Yue Shen, Shengbo Jin, Mingzhu Li

**Affiliations:** ^1^ Liaoning University of Traditional Chinese Medicine, Shenyang, Liaoning, China; ^2^ Cancer Hospital of China Medical University, Liaoning Cancer Hospital & Institute, Shenyang, Liaoning, China; ^3^ Dalian Medical University, Dalian, Liaoning, China; ^4^ China Medical University, Shenyang, Liaoning, China; ^5^ Affiliated Hospital of Liaoning University of Traditional Chinese Medicine, College of Liaoning Chinese Traditional Medicine, Shenyang, Liaoning, China

**Keywords:** oxaliplatin, neuropathic pain, CINP animal model, chemotherapy, applications

## Abstract

Oxaliplatin, a third-generation platinum-based chemotherapeutic agent, has shown substantial efficacy in cancer treatment. However, its associated side effects, particularly chemotherapy-induced peripheral neuropathic pain (CIPNP), continue to challenge cancer survivors globally. Clinically, it frequently presents as numbness, coldness, and discomfort in the limbs and extremities. Duloxetine is advised for analgesic purposes. Despite its clinical relevance, both the application methods and the underlying mechanisms of oxaliplatin-induced CINP warrant further investigation. Consequently, more precise animal models are needed to explore the mechanisms and progression of this condition. This review consolidates recent advancements in rat and mouse models of oxaliplatin-induced CINP, with the aim of enhancing modeling success rates and developing models that more accurately mirror disease progression. Such models are essential for advancing clinical research and drug development.

## 1 Introduction

The increasing incidence of malignant tumors has led to a growing reliance on chemotherapy drugs as first-line antitumor agents. Platinum-based drugs are the main chemotherapeutic agents for treating Colorectal cancer (CRC) (Cartwright and Cunningham, 2017). Statistics show that approximately 65%–98% of chemotherapy patients develop peripheral neuropathy following oxaliplatin treatment (Cavaletti and Marmiroli, 2020). Oxaliplatin, a third-generation platinum-based cytotoxic derivative, is primarily used to treat advanced colorectal cancer and is considered to have a more favorable safety profile compared to other platinum-based agents (Sałat et al., 2019a). However, its side effects significantly compromise patients’ quality of life. Oxaliplatin therapy is primarily linked to three dose-limiting toxicities: neurotoxicity (notably oxaliplatin-induced peripheral neuropathy, OIPN), myelosuppression, and gastrointestinal problems (Marmiroli et al., 2017). These adverse effects significantly influence therapy success, especially considering the characteristic dose-dependent course of OIPN that often requires treatment alteration. Oxaliplatin exhibits a mechanism of action that is fundamentally different from traditional platinum-based chemotherapeutics, as its cumulative neurotoxicity shows clinically meaningful reversibility after treatment discontinuation, a neuroprotective characteristic absent in cisplatin-class drugs (Starobova and Vetter, 2017).

Chemotherapy-induced neuropathic pain (CINP)is a debilitating, dose-limiting toxicity of cancer chemotherapy. This neuropathy not only markedly diminishes quality of life but also often compels patients to modify or discontinue their treatment, presenting a significant clinical challenge (Mantyh, 2006).

Despite its clinical relevance, the mechanisms underlying CINP remain poorly understood. Acute OIPN symptoms may present as dyspnea and dysphagia, whereas chronic OIPN clinical signs encompass distal sensory abnormalities, decreased proprioception, and diminished deep tendon reflexes (Marmiroli et al., 2017). However, the mechanisms underlying CIPNP (chemotherapy-induced peripheral neuropathy) have not yet been fully elucidated. OIPN is associated with alterations in voltage-gated Na^+^ channels, as well as K^+^ channels, Ca^2+^ channels, and transient receptor potential (TRP) channels. Abnormalities in voltage-gated Na^+^ channels lead to prolonged channel opening and excessive excitability of dorsal root ganglion (DRG) sensory neurons (Webster et al., 2005). These changes induce ectopic discharges, resulting in the typical symptoms of oxaliplatin-induced sensory abnormalities. Prior research by academics has demonstrated that OXA-induced cold hyperalgesia increases the production of TRPA1 protein on TRP channels. CINP induced by OXA intensifies oxidative stress, thus modulating TRPA1 and aggravating CINP symptoms (Lim et al., 2010). The principal factors contributing to chronic OIPN include nuclear DNA damage, mitochondrial impairment, excessive oxidative stress, and activation of glial cells (Staff et al., 2019). Furthermore, mitochondrial impairment significantly contributes to CINP produced by OXA. *In vitro* findings indicate that the mitochondrial structure and function of rat neural cells exposed to OXA are modified (Bobylev et al., 2018). The activation of neuroimmune responses is a significant role in the development of CINP. The aberrant activation between neurons and glial cells is essential for neuropathic pain.

According to the 2020–2021 clinical practice guidelines of the American Society of Clinical Oncology (ASCO), no preventive or therapeutic strategies have been definitively established for CINP(11). As such, the selection of suitable animal models for research is of paramount importance. Current mechanistic studies commonly employ rat or mouse models of CINP, yet the absence of clear classifications and standardized evaluation criteria hinders the optimal use of these models, thereby affecting the reliability and validity of research outcomes. While various therapeutic approaches exist for chronic pain, certain researchers have performed pain-relief tests on mice utilizing an individual intra-abdominal injection of oxaliplatin (3 mg/kg) in conjunction with milnacipran. The mechanical pain in these animals peaked 10 days post-injection. Nevertheless, the mechanical pain threshold in the mice was diminished following the administration of milnacipran (Hershman et al., 2014). In addition, ASCO only recommends duloxetine for treatment. Nevertheless, the aforementioned therapies still have many shortcomings and deficiencies. Therefore, it is urgent to seek more perfect animal models for clinical treatment.

This paper discusses the methodologies for establishing rat and mouse CINP models induced by oxaliplatin, along with specific evaluation techniques and their applications in clinical research. The objective is to provide more precise and effective approaches for advancing CINP research.

## 2 Main methods for establishing oxaliplatin-induced neuropathic pain models in rats and mice

The common methods for establishing rat and mouse CINP models include intraperitoneal and intravenous injections. Both techniques are relatively safe and straightforward, with intraperitoneal injection being the predominant method used globally for CINP modeling (Al Moundhri et al., 2013). Clinical trials indicate that oxaliplatin-induced peripheral neuropathy (OIPN) is a cumulative adverse event, typically manifesting when the total oxaliplatin dose exceeds 750–850 mg/m^2^ (Soveri et al., 2019; de Gramont et al., 2023). Prior research indicates that significant symptoms of oxaliplatin-induced chemotherapy-induced peripheral neuropathy during the acute phase correlate with similarly severe symptoms transitioning to the chronic phase in the initial treatment cycle (Pachman et al., 2015). Furthermore, the manifestations of oxaliplatin-induced CINP endure for an extended period. Post-chemotherapy, patients’ neuropathy persists and exacerbates due to the continued accumulation of oxaliplatin in the dorsal root ganglion (DRG) (Staff et al., 2019).

Rat CINP models are generally classified into acute and chronic types. Acute models involve a single or short-term high-dose injection of oxaliplatin, while chronic models utilize prolonged low-dose injections. Varying the injection dose and duration can induce different degrees of nerve damage. Research has demonstrated that a single intraperitoneal injection of oxaliplatin (6 mg/kg) reliably induces peripheral neuropathic pain in rats, primarily characterized by mechanical and cold allodynia (Baek et al., 2024).

Post-oxaliplatin treatment, rats exhibit several behavioral changes, including reduced food intake, weight loss, or stunted weight gain. Other signs may include limb muscle atrophy, increased paw licking, and paw lifting. Additionally, mechanical pain thresholds and cold/heat pain thresholds are altered. These behavioral manifestations serve as critical indicators for assessing the success of model establishment.

The table below offers a detailed summary of commonly employed acute and chronic modeling methods, along with their associated behavioral characteristics and safety assessments.

## 3 Methods for model evaluation

Oxaliplatin-induced neuropathic pain in clinical settings is characterized primarily by limb numbness, cold pain, and sensory disturbances. Approximately 80% of chemotherapy patients develop chemotherapy-induced peripheral neuropathy (CINP), with 60% continuing to experience symptoms 3 months post-treatment (Hu et al., 2019). These clinical manifestations are also evident in rat and mouse CINP models. Consequently, model evaluation typically involves assessing mechanical pain thresholds, mechanical allodynia, and alterations in cold and heat pain thresholds, along with cold/heat allodynia. The following are the evaluation methods most frequently used in recent studies.

### 3.1 Measurement of mechanical pain threshold

#### 3.1.1 Von Frey filament pain threshold test

The experimental procedure is as follows: Mice are placed in a raised plexiglass chamber (10 × 5 × 5 cm) with a metal mesh floor. Prior to the experiment, the mice are allowed to acclimate for 15 min. A metal filament is used to apply progressively increasing mechanical pressure to the central region of the plantar layer of the hind paw until a clear paw withdrawal response is elicited. The response threshold, recorded in grams (g), represents the mechanical withdrawal threshold (MWT), defined as the minimum pressure required to provoke a rapid and pronounced paw withdrawal. The pressure stimulus is applied to each hind paw every 30 s. Each experiment consists of five measurements, with the final result being the average of these measurements. This experimental design ensures both accuracy and reproducibility of the data (Le et al., 2021). Keisuke et al. (Mine et al., 2022) conducted a study investigating the mitigation of oxaliplatin-induced peripheral neuropathy with omeprazole, employing mechanical allodynia assessments in rats post-drug application. Prior to the test, the rats were positioned on a metal mesh for 30 min to acclimatize. The rats’ hind paws were stimulated from beneath the mesh for 6 s each time. The “up-and-down” approach was employed for measurement, and the intensity of the Von Frey filament that provoked an escape response in the rats was documented as the escape response threshold.

#### 3.1.2 Randall-Selitto paw withdrawal test

The test employs a Basile Algesimeter (Chicago, Illinois) to assess the paw pressure threshold in rats. During the procedure, rats are placed in a Perspex cylindrical restrainer with sliding doors, allowing the hind limbs to extend freely through a ventilation hole. Pain thresholds are measured before and after drug administration, with readings taken every 5 min, resulting in four measurements. The final pain threshold is determined as the average of the last three measurements. To prevent tissue damage, the maximum withdrawal pressure is limited to 200 g. This approach is scientifically structured to ensure the reliability and consistency of the results (Joseph et al., 2008; Mori et al., 2014).

#### 3.1.3 Dynamic Aesthesiometer Test

The dynamic aesthesiometer (Model 37,450, Ugo Basile Biological Instruments, Comerio, Italy) assesses the progression of mechanical allodynia by applying a linearly increasing mechanical force. A sharp metal filament (0.5 mm in diameter) is placed beneath the rat’s hind paw, and intermittent pressure is gradually applied, reaching 15 g over 15 s to evoke a distinct paw withdrawal response, which serves as the measure for the mechanical pain threshold. Each hind paw is tested three times, with the average result taken as the final measurement. The maximum stimulation duration is 15 s, after which the device automatically ceases the mechanical stimulus. During right hind paw plantar testing, pressure is applied at a rate of 0.5 g/s, with a maximum of 5 g. If no paw withdrawal response occurs within 30 s, the test is manually halted, and the probe is retracted. Each rat undergoes five repeated measurements, ensuring at least 10-min intervals between tests. The average of these five measurements is used for analysis to enhance data accuracy and consistency. Two-way ANOVA followed by Sidak’s multiple comparison test (Marmiroli et al., 2017; Gould et al., 2021). The behavioral experiments related to Ballarini et al. (Ballarini et al., 2022)’s study on oxaliplatin neurotoxicity in mice involved the application of a pointed metallic filament, measuring 0.5 mm in diameter, to the plantar surface of the hind paw to exert increasing punctate pressure. The pressure at which the mice exhibited a distinct hind-paw withdrawal reaction was documented, and the stimulus was automatically ceased upon reaching the maximum duration of 15 s.

### 3.2 Measurement of mechanical allodynia

#### 3.2.1 Von Frey filament test

The Von Frey filaments (0.6, 1.0, 1.4, 2.0, 4.0, 6.0, 8.0, 10.0, 15.0, 26.0, and 60 g) are used in combination with an electronic device (Bioseb, France Model: BIO-VF-M) for mechanical allodynia assessment via the Von Frey test. Mice (15–21 per test) are individually placed in a compartmentalized room with a mesh floor, allowing acclimation for at least 30 min prior to testing (Aloi et al., 2023). The Von Frey stimulus is applied to the plantar surface of the rats’ paws. A threshold is determined if at least three of five applications result in a response, with a cutoff of 60 g. During testing, mice are housed in transparent plastic boxes with metal mesh floors, ensuring full contact with the plantar surface. Prior to testing, mice are allowed to acclimate for at least 15 min. A series of nine Von Frey filaments, with logarithmically increasing stiffness (0.023–3.630 g), is used, starting with a 0.407-g filament (Akaberi et al., 2024; Toyama et al., 2017). The mechanical withdrawal threshold was evaluated utilizing the “up-and-down” strategy as delineated by Llorian-Salvador et al. (Llorián-Salvador et al., 2016). One of the mouse’s hind paws (left or right) was randomly chosen for examination. Should the mouse exhibit a positive response, such as elevating or licking the paw, a finer Von Frey filament was utilized in the subsequent test; otherwise, a thicker filament was employed. Six measurements were conducted for each mouse. The 50% response threshold was calculated using the following formula: 50% threshold (g) = (10^Xf + kd)/10,000. The correction factor k was derived from the response patterns in Dixon’s calibration table, whereas d indicated the average distance between filament diameters. The stated threshold value was the mean of the thresholds derived from the left and right hind paws (Marcotti et al., 2023).

#### 3.2.2 Randall-Selitto test

An analgesimeter (Ugo Basile, Varese, Italy) is used to assess the nociceptive threshold by applying gradually increasing pressure to the dorsal or plantar surface of the hind paw or tail. The animal is restrained in a sling, with support provided by a towel, plastic cone, or cylinder, allowing access to the hind paw. A sharp probe applies pressure between its tip and a flat surface, which is increased at a constant rate until a nociceptive response, such as vocalization or paw withdrawal, is elicited. The nociceptive threshold is recorded in grams (g). A blunt conical probe targets a small area on the dorsal surface of the rat’s hind paw, and pressure is applied until the threshold is reached. To prevent tissue damage, the maximum pressure is limited to 200 g. The procedure involves minimal restraint of the animals (Deuis et al., 2017; Bonifacino et al., 2022).

### 3.3 Measurement of cold allodynia

#### 3.3.1 Cold plate test

Rats and mice are placed on a cold plate maintained at 4°C, and the latency to paw withdrawal is measured by observing behavioral responses, such as jumping, paw licking, or paw lifting. The maximum observation time is 30 s. The cold plate test (Model 35,100 - Hot/Cold Plate, Ugo Basile) evaluates the cold pain threshold, utilizing a cylindrical plexiglass chamber and a thermostatic plate that generates variable temperatures. Mice are allowed free movement on the plate set to 4°C. Abnormal behaviors, including tail movements, sudden jumps, and other pain indicators, are recorded during a 5-min trial. To prevent potential tissue damage, a 60-s cutoff time is established. Animals failing to respond within this period are removed from the apparatus, with their latency recorded as 60 s (Sałat et al., 2019a; Marmiroli et al., 2017; Tsubaki et al., 2018).

#### 3.3.2 acetone test

##### 3.3.2.1 Cold pain sensitivity test with acetone spray

Cold allodynia in the hind paw is evaluated by gently spraying an acetone solution at predetermined intervals. A total of 20 μL of acetone is drawn into a syringe and swiftly applied to the plantar surface of the mouse’s paw, ensuring no direct contact with the skin. Both the left and right hind paws are tested, and the average response within 60 s is recorded (Toma et al., 2017). A behavioral investigation examining the preventative effect of a superoxide dismutase modulator on oxaliplatin-induced neuropathy demonstrated that cold sensitivity in mice was assessed following daily high-dose injection. The cold plate test was conducted at an ideal temperature of 2°C ± 0.2°C, and the mice’s responses were monitored within a 5-min interval, with the frequency of vigorous lifts of one or both hind paws recorded as an indicator of cold sensitivity. The cold plate test was performed at baseline prior to treatment, on day 5 of treatment, and on day 10 of each cycle. The results were presented as the mean ± standard deviation of the observers’ counts and were statistically examined using the Mann–Whitney test (Guillaumot et al., 2019).

##### 3.3.2.2 Acetone drip test

The acetone drip test is another applicable method, where acetone is dripped onto the left hind paw of the animal every 3 min throughout the experiment, for a total of five applications. Results are recorded as positive or negative based on the presence or absence of a leg-lifting response (Alaei et al., 2023). Vincenzo et al. (Aloi et al., 2023) employed a greater concentration of acetone solution for application via spraying. Thirty minutes before to the official commencement of the trial, the mice designated for testing were situated in a distinct compartment featuring eight metal mesh floors. Subsequently, 50 μL of acetone solution was administered via spray to one of the rear paws of the mice. Subsequently, two researchers separately evaluated the behavior of the mice. The evaluations from the two researchers were quite consistent. The mean of the scores from the two researchers was utilized for statistical analysis.

##### 3.3.2.3 Acetone injection test

Prior to testing, the rat is placed in a plastic box with a metal mesh bottom for 15 min of acclimation. Using a 0.5 mL syringe with a 26-gauge needle, acetone is injected from beneath the mesh floor onto the ventral surface of one hind paw. The rat’s reaction is monitored for 20 s, and if no response is observed within this period, it is recorded as no response. If a response occurs, an additional 40 s of monitoring is performed, resulting in a total observation time of 1 min from the initial acetone application. Acetone is applied alternately to each hind paw twice, with a 5-min interval between applications (Matsuura et al., 2021). In Karen’s study (Álvarez-Tosco et al., 2024),to evaluate cold allodynia, a 200 µL droplet of acetone was administered to the plantar surface of both hind paws of the mice on days 0, 1, 3, and five of the experiment using an insulin-type syringe. The duration of time the mice engaged in twitching, biting, or licking the stimulated paw was monitored and documented over a span of 2 min.

#### 3.3.3 Cold water tail immersion test

The latency to tail withdrawal in a cold-water bath is used as an indicator of anti-nociceptive effects. The rat’s tail is immersed in water maintained at 4°C or 10°C, and the time from immersion to withdrawal is recorded. The procedure is repeated 3 to 5 times with 5-min intervals between measurements, and the average latency is calculated. To prevent tail tissue damage, the maximum immersion time is capped at 15 s (Alaei et al., 2023; Ling et al., 2007). Chen et al. (Chen R. et al., 2024) utilized a comparable approach for detection. The researchers submerged the tails of rats in a cold water bath at 4°C and performed assessments on days 0, 3, 6, 9, 12, and 15, monitoring and documenting the duration until the animals voluntarily retracted their tails. Each test was conducted with a 5-min interval, comprising a total of three trials. The mean of these three recorded numbers was considered the final result. An upper time restriction of 15 s was established for the experiment to avert tissue harm from low temperatures.

#### 3.3.4 Cold plantar test

Dry ice is crushed into fine powder and packed into a modified 3 mL syringe. The open end of the syringe is pressed against a flat surface, and the plunger is used to compress the dry ice into flat, dense pellets approximately 1 cm in diameter. During the experiment, the tip of the dry ice pellet is extended from the syringe and applied to the central region of the rat’s hindfoot with light and steady pressure, avoiding distal joints. Full contact between the paw and the experimental surface is ensured. A stopwatch is used to record the time from the application of pressure to the paw withdrawal response, which is used to assess the cold pain threshold. Stable pressure application is maintained throughout to ensure the accuracy and reproducibility of the results (Ippolito et al., 2024).

### 3.4 Heat allodynia test

#### 3.4.1 Hot plate test

For heat allodynia, rats are placed on a hot plate maintained at 50°C, and the latency to licking the left hind paw or jumping is recorded. The test is conducted using a hot plate device featuring a 19 cm diameter metal screen and a 30 cm high plexiglass enclosure. The device is electrically heated to a constant temperature of 50°C ± 1°C and is connected to a timer and thermostat. The time from the start of the test to the animal’s heat pain response, such as forelimb licking or jumping, is recorded as the heat pain reaction time. To prevent injury, the maximum response time is set at 30 s (Alaei et al., 2023; Kukkar et al., 2013). Based on the aforementioned experimental methods, Chen et al. (Chen R. et al., 2024) positioned the rats on a heated plate enclosed with an acrylic lid. Following a 1-min acclimatization period, the detection commenced. Subsequently, the initial occurrence of foot-licking behavior in the rats was documented. The test was administered every 5 min, repeated three times, and the average of these records was computed, with a maximum duration of 60 s for each test.

#### 3.4.2 Thermal radiation method

##### 3.4.2.1 Tail flick test with thermal radiation

The tail flick test, using equipment from Ugo Basile (Milan, Italy), assesses the thermal pain threshold in rats. Infrared heat is applied to the tail, and the nociceptive threshold is automatically measured. The operator activates the stimulation device, and when the rat perceives pain and flicks its tail, a sensor detects the response, stops the timer, and turns off the heat lamp. The tail flick test can involve radiant heat stimulation or immersion of the tail in water baths set at 46°C–52°C. The time required to induce tail flicking or withdrawal is recorded (Toyama et al., 2017; Chen X. et al., 2024).

##### 3.4.2.2 Thermal radiation test on hind paws

Prior to modeling, the thermal pain threshold of the rats is measured. They are placed in a plexiglass box on a glass platform for 20 min before testing. The device is adjusted to ensure the baseline thermal paw withdrawal latency is between 10 and 12 s. If a rat does not exhibit a response, such as paw lifting or licking, within 15 s, it is considered non-responsive, and the power is automatically shut off to prevent injury. Each hind paw is tested three times, with 10-min intervals between measurements (Yu et al., 2021).

#### 3.4.3 Hot water bath rat tail immersion test

Thermal pain sensitivity was evaluated by immersing the last 3 cm of the rat’s tail tip into water baths maintained at either low (10°C) or high (42°C) temperatures. The latency of the tail-flick reflex was measured as an indicator of thermal nociception (Li et al., 2018; Casadei et al., 2022).

#### 3.4.4 Hargreaves experiment

The animal was placed in an enclosed glass chamber, and a thermal stimulus was applied to the plantar surface of the hind paw using a radiation or infrared source positioned at a fixed distance. When the hind paw retracted in reply to the thermal stimulus, this was recorded as a response. The Hargreaves thermal thresholds and the latency to elicit the retraction response were measured and documented (Chen X. et al., 2024). Following a 15-min acclimatization period for the rats, a 50-W halogen lamp was employed to irradiate the plantar areas of both hind paws. The technique was conducted three times, with a 5-min gap between each experiment. A cutoff period of 33 s was established, and the average withdrawal delay of the paw was determined as the final value (Mojadadi et al., 2025).

### 3.5 Assessment of motor coordination

#### 3.5.1 Rotarod test

Before the experiment, animals undergo adaptive training on the rotarod instrument for three successive days. The rotarod operates at a fixed speed of 18 revolutions per minute (rpm). During each training session, the mice are placed on the rotating rod for 3 min per session, with no limit on the number of sessions. The formal experiment is conducted 24 h subsequent to the ultimate training session. During the experiment, mice are tested at rotarod speeds of 6 rpm, 18 rpm, and 24 rpm after receiving experimental drugs or control solvents. A mouse is considered to have motor dysfunction if it fails to stay on the rotarod for at least 1 min. The results are expressed as the average time spent on the rotarod, serving as an indicator of motor function (Sałat et al., 2015).

A rotarod treadmill (Ugo Basile, Milan, Italy) is used to assess the neuromuscular coordination of rats in control and treatment groups. The time from the rat stepping onto the rotarod to falling off is recorded using the device’s built-in timer. This time is considered the motor performance time (Alaei et al., 2023).

#### 3.5.2 Grip strength test

For the motor strength grip test, the forepaws of each rat are placed on the grip bar, and the tension gauge is zeroed. The experimenter gently pulls the rat’s tail backward until it releases the bar, recording the reading on the tension gauge. This procedure is repeated four times for each rat (Zhou et al., 2019).

## 4 Discussion

**FIGURE 1:**
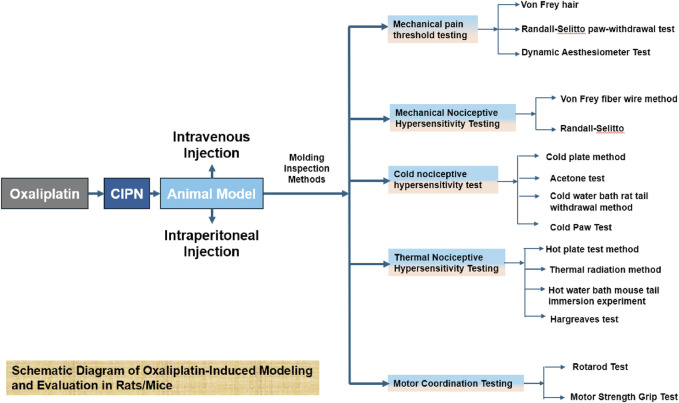
This figure provides a detailed illustration of the modeling process and subsequent evaluation methods for oxaliplatin-induced effects in rats and mice.

**FIGURE 2 F2:**
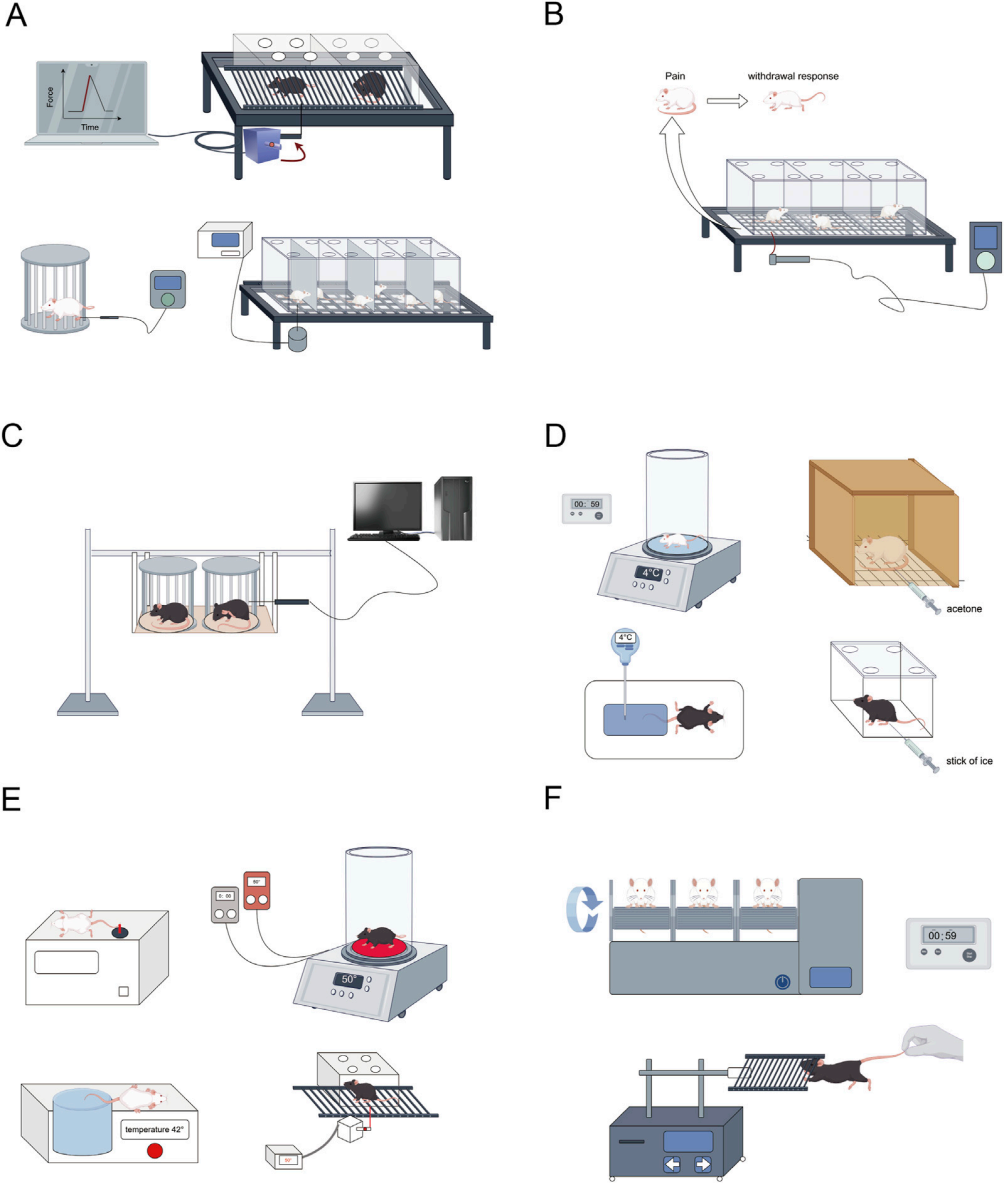
Conduct relevant behavioral and other tests on the successfully modeled rats. **(A)** Mechanical pain threshold testing (Von Frey hair, Randall-Selitto paw-withdrawal test, Dynamic Aesthesiometer Test); **(B,C)** Mechanical nociceptive hypersensitivity testing (Von Frey fiber wire method, Randall-Selitto); **(D)** Cold nociceptive hypersensitivity test (Cold plate method4°C, acetone test, Cold water bath rat tail withdrawal method, Cold paw test); **(E)** Thermal nociceptive hypersensitivity testing (Hot plate test method 50°C ± 1°C, Thermal radiation method, Hot water bath mouse tail immersion experiment, Hargreaves test); **(F)** Motor coordination testing (Rotarod test, Motor strength grip test). This figure was created using Figdraw.

CINP is predominantly characterized as a sensory neuropathy with symmetrical symptoms. Common clinical manifestations include numbness, proprioception loss or impairment, tingling, pricking sensations, and hyperalgesia or allodynia, often affecting the hands and feet in a “stocking-and-glove” distribution pattern (Park et al., 2013). Oxaliplatin-associated CINP presents acutely and exacerbates with successive chemotherapy cycles (Pachman et al., 2016). It is estimated that 65%–98% of chemotherapy patients receiving oxaliplatin develop peripheral neuropathy (Cavaletti and Marmiroli, 2020). Currently, there is no definitive treatment for CINP in Western medicine. The 2020–2021 guidelines from the ASCO recommend duloxetine for pain management (Loprinzi et al., 2020), though its limited efficacy and notable side effects underscore the need for alternative therapeutic strategies. Additional approaches, such as minimally invasive interventional treatments, physical therapy, and external therapies like acupuncture, have been explored for treating CINP(51). We have encapsulated the applications of rats and mice in simulating CINP, detailing specific methodologies, safety protocols, injection dosages, and additional considerations. The table delineates the modeling techniques for the CINP acute model in rats (Table 1), alongside its applicability across several rat types (Table 2), mice (Table 3), and the injection methodologies for both rats and mice (Table 4)(Figures 1,2, [Fig F2]).

**TABLE 1:** Rat acute CINP models under different doses and injection methods.

Modeling methods	Injecting drugs	Total drug dose	Pain behavior	Rat species	Safety	Applicable research areas	References
5 mg/kg, injected once	i.p	5 mg/kg	+MA, +CA	SD male rats	Deathless description	Mechanism research	Matsuura et al. (2021)
10 mg/kg, injected once	i.p	10 mg/kg	+MA, +HA	CD1 male mice	Deathless description	Curative effect research	Furgała-Wojas et al. (2020)
10 mg/kg, injected once	i.p	10 mg/kg	+MA, +motor deficit	CD1 male mice	Deathless description	Developmental research	Sałat et al. (2018)
10 mg/kg, injected once	i.p	10 mg/kg	+CA	CD-1 mice	Deathless description	Developmental research	Furgała et al. (2018)
6 mg/kg, injected once	i.p	6 mg/kg	+MA, +CH	SD male rats	Clearly document that no deaths are recorded	Mechanism research	Li et al. (2018)
6 mg/kg, injected once	i.p	6 mg/kg	+MA	SD male rats	Deathless description	Mechanism research	Huang et al. (2018)
10 mg/kg, injected once	i.p	10 mg/kg	+MA	CD-1 mice	Deathless description	Developmental research	Rapacz et al. (2018a)
10 mg/kg, injected once	i.p	10 mg/kg	+MA	CD1 male mice	Deathless description	Developmental research	Rapacz et al. (2018b)
10 mg/kg, injected once	i.p	10 mg/kg	+MA	CD1 male mice	Deathless description	Developmental research	Rapacz et al. (2016)
10 mg/kg, injected once	i.p	10 mg/kg	+MA, +CH, +motor deficit	CD1 male mice	Deathless description	Curative effect research	Sałat et al. (2017)
3 mg/kg, injected once	i.p	3 mg/kg	+MA	C57Bl/6 male mice	Deathless description	Developmental research	Moreira et al. (2017)
5 mg/kg, injected once	i.v	5 mg/kg	+MA, +CH, +CA	BALB/c male mice	Deathless description	Developmental research	Canta et al. (2020)
10 mg/kg, injected once	i.p	10 mg/kg	+MA, +CA	CD-1 male mice	Deathless description	Curative effect research	Sałat et al. (2019b)
3 mg/kg, injected once	i.p	3 mg/kg	+MA, +CH, +CA	C57BL/6 mice or TRPA1-deficient mice	Deathless description	Mechanism research	Trevisan et al. (2013)
6 mg/kg, injected once	i.p	6 mg/kg	+MA, +CH, +CA	BL/6 mice, WldS and Sarm1−/− male mice	Deathless description	Developmental research	Gould et al. (2021)
10 mg/kg, injected once	i.p	10 mg/kg	+CH	CD-1 male mice	Deathless description	Curative effect research	Sałat et al. (2019a)
3 mg/kg, injected once	i.p	3 mg/kg	+MA	SD male rats	Deathless description	Mechanism research	Illias et al. (2018)
2 mg/kg, injected once	i.v	2 mg/kg	+MA	SD male and female rats	Deathless description	Mechanism research	Bonet et al. (2022)
2 mg/kg, injected once	i.v	2 mg/kg	+MA	SD male and female rats	Deathless description	Mechanism research	Staurengo-Ferrari et al. (2022)
2 mg/kg, injected once	i.v	2 mg/kg	+MA	SD male rats	Deathless description	Mechanism research	Araldi et al. (2024)
6 mg/kg, injected once	i.p	6 mg/kg	+CA	SD rats	Deathless description	Curative effect research	Baek et al. (2024)
6 mg/kg, injected once	i.p	6 mg/kg	+MA, +CA	SD rats	Deathless description	Curative effect research	Choi et al. (2019)
10 mg/kg, injected once	i.p	10 mg/kg	+MA	SD male rats	Deathless description	Developmental research	Warren et al. (2024)

Note. i. p. Intraperitoneal injection. i. v. Intravenous injection. CA., Cold allodynia; CH., Cold hyperalgesia. HA. Heat allodynia. HH., Heat hyperalgesia; MA., Mechanical allodynia; MH., Mechanical hyperalgesia. +. This behaviour was assessed as pain-like following oxaliplatin administration. This behaviour was assessed as insignificant following oxaliplatin administration. (The same in the following tables).

**TABLE 2:** Rat CINP models with different doses and injection methods.

Modeling methods	Injecting drugs	Total drug dose	Pain behavior	Rat species	Safety	Applicable research areas	References
4 mg/kg, injected on days 0, 2, 4	i.p	12 mg/kg	+MA、+HA	SD male and female rats	Deathless description	Developmental research	Noya-Riobó et al. (2023)
4 mg/kg, injected once per day for 5 days	i.p	20 mg/kg	+MA	SD male and female rats	Deathless description	Mechanism research	Dong et al. (2022)
2.4 mg/kg, injected on days 1–3, 6–10, and 13–15	i.p	26.4 mg/kg	+MA, +MH, +CA	SD male rats	Deathless description	Curative effect research	Bonifacino et al. (2022)
2 mg/kg, injected on days 1, 2, 3, 4, 5	i.p	20 mg/kg	+MA, +MH, +HA, +CA	SD female rats	Deathless description	Mechanism research	Li et al. (2022)
6 mg/kg, injected on days 1,3, 5, 7	i.p	24 mg/kg	+HH	SD male rats	Deathless description	Mechanism research	Yu et al. (2021)
4 mg/kg, injected once per day for 5 days	i.p	20 mg/kg	+MA	SD male and female rats	Deathless description	Mechanism research	Huang et al. (2021)
2 mg/kg, injected once	i.v	2 mg/kg	+MH	SD male rats	Deathless description	Developmental research	Staurengo-Ferrari et al. (2023)
4 mg/kg, injected twice a week for 4 weeks	i.p	32 mg/kg	+MH	SD male rats	Deathless description	Mechanism research	Maruta et al. (2019)
2.4 mg/kg, 3.2 mg/kg or 4.0 mg/kg, injected twice weekly for4.5 weeks	i.p	21.6 mg/kg, 28.8 mg/kg, or 36 mg/kg	+MH, +CH	SD male rats	Deathless description	Developmental research	Li et al. (2019)
4 mg/kg, injected on days 1, 2, 8, 9, 15, 16, 22, and 23	i.p	32 mg/kg	+MH, +CH	SD male rats	Deathless description	Curative effect research	Zhou et al. (2019)
4 mg/kg, injected twice a week for 4 weeks	i.p	32 mg/kg	+MH, +CH	SD male rats	Deathless description	Mechanism research	Liu et al. (2018)
2.4 mg/kg, injected daily for 2 weeks	i.p	33.6 mg/kg	+MH, +HH	SD male rats	Deathless description	Developmental research	Chelini et al. (2017)
6 mg/kg, injected daily for five consecutive days	i.p	30 mg/kg	+MA, +CA	SD male rats	Deathless description	Curative effect research	Jung et al. (2017)
4 mg/kg, injected twice a week for 4 weeks	i.p	36 mg/kg	+MH	Wistar female rats	Deathless description	Curative effect research	Deng et al. (2016)
3 mg/kg, injected on days 0, 2, 4, 6	i.p	12 mg/kg	+MA, +motor deficit	SD male and female rats	Deathless description	Mechanism research	Illias et al. (2022)
6 mg/kg, injected once per day for 6 days	i.p	36 mg/kg	+CA	Wistar male rats	Clearly document that no mice deaths are recorded	Mechanism research	Álvarez-Tosco et al. (2024)
5 mg/kg, injected on days 1,4	i.p	10 mg/kg	+MH, +CH	SD male and female rats	Deathless description	Curative effect research	Ippolito et al. (2024)
2 mg/kg, injected 5 consecutive days	i.p	10 mg/kg	+MA, +MH	SD male and female rats	Deathless description	Curative effect research	Wagner et al. (2024a)
6 mg/kg, injected on days 1, 3, 5, 7	i.p	24 mg/kg	+MH, +CH, +HH	SD male rats	Deathless description	Mechanism research	Shao et al. (2023)
4 mg/kg, injected on days 0, 2, 4	i.p	12 mg/kg	+MH, +CH	SD male and female rats	Deathless description	Developmental research	Shao et al. (2023)
4 mg/kg, injected once per day for 5 consecutive days	i.p	20 mg/kg	+MH	SD male and female rats	Deathless description	Mechanism research	Deng et al. (2020)
2 mg/kg, injected for five consecutive days	i.v	10 mg/kg	+MA, +MH	SD male and female rats	Deathless description	Curative effect research	Wagner et al. (2024b)

**TABLE 3:** Mice CINP models with different doses and injection methods.

Modeling methods	Injecting drugs	Total drug dose	Pain behavior	Mice species	Safety	Applicable research areas	References
3 mg/kg, 5 consecutive days, followed by 2 days of rest, for two cycles	i.p	30 mg/kg	+MA	C57BL/6J male mice	Deathless description	Mechanism research	Dai et al. (2024)
low-dose (0.3 mg/kg) or high-dose oxaliplatin (3 mg/kg) for 5 consecutive days, followed by 5 days of rest, followed by a second cycle of five daily injections	i.p	3 mg/kg 30 mg/kg	+MA, +CA	C57BL/6J and BALB/cJ male and female mice	Deathless description	Experimental Research	Warncke et al. (2021)
3 mg/kg, three times per week for 4 weeks	i.p	36 mg/kg	+HH	C57BL/6 WT mice	Deathless description	Mechanism research	Jang et al. (2021)
2.4 mg/kg, 5 consecutive days each week for 2 weeks	i.p	24 mg/kg	+CA	CD-1 male mice	Deathless description	Curative effect research	Micheli et al. (2023)
2.4 mg/kg, injected 2 weeks	i.p	33.6 mg/kg	+HA	CD-1 male mice	Deathless description	Mechanism research	Crocetti et al. (2022)
2.4 mg/kg, injected 2 weeks	i.p	33.6 mg/kg	+MA, +MH, +HA	CD-1 male mice	Deathless description	Mechanism research	Micheli et al. (2021)
2.4 mg/kg, 5 consecutive days every week for 2 weeks	i.p	24 mg/kg	+MH, +HH	CD-1 mice	Deathless description	Developmental research	Micheli et al. (2020)
30 mg/kg, injected once	i.p	30 mg/kg	+CH	STZ mice	Deathless description	Developmental research	Zaręba et al. (2020)
10 mg/kg, injected once per week for 3 weeks	i.p	30 mg/kg	+MA, +HH	BALB/c male mice	Deathless description	Mechanism research	Toyama et al. (2018)
6 mg/kg, injected daily for a continuous 5 days	i.p	30 mg/kg	+MH, +CH	C57BL/6 male mice	Deathless description	Curative effect research	Li et al. (2015)
3 mg/kg, injected daily administration for 5 days, followed by 5 days of rest, for 2 weekly cycles	i.p	30 mg/kg	+MA, +CA	ICR male mice	Deathless description	Curative effect research	Zhou et al. (2016)
5 mg/kg, injected daily for five consecutive days	i.p	25 mg/kg	+MA, +CA	C57BL/6 male mice	Deathless description	Curative effect research	Le et al. (2021)
3 mg/kg, injected daily administration for 5 days, followed by 5 days of rest, for 2 weekly cycles	i.p	30 mg/kg	+MA, +CH	BALB/c male and female mice	Deathless description	Curative effect research	Rukh et al. (2020)
3 mg/kg, injected daily administration for 5 days	i.p	15 mg/kg	+MH, +CH	C57BL/6 mice	Deathless description	Curative effect research	Ho et al. (2025)
6 mg/kg, every 3 days over 6 days	i.p	12 mg/kg	+MA, +CA	Swiss male and female mice	Deathless description	Curative effect research	Mroué et al. (2024)
5 mg/kg, injected on days 0, 2, 4, 6	i.p	20 mg/kg	+MH	C57BL/6J and DEREG male mice	Deathless description	Developmental research	Makker et al. (2017)
2 mg/kg, injected twice a week for 2 weeks	i.p	8 mg/kg	+MA, +CA+, HA,+motor deficit	Balb-c male mice	Deathless description	Mechanism research	Bilgin et al. (2025)
10 mg/kg, injected on days 0, 2	i.p	20 mg/kg	+MA, +HH	Swiss male mice	Clearly document that no mice deaths are recorded	Developmental research	Reis et al. (2025)
5 mg/kg, injected twice per week for 5 weeks	i.p	50 mg/kg	+MA, +CH	C57BL/6 male mice	Deathless description	Developmental research	Kuroda et al. (2024)
3 mg/kg 3 mg/kg, injected for 5 consecutive days or 2.8 mg/kg injected into mice twice a week for 4 weeks	i.p	15 mg/kg or 28 mg/kg	+MH, +CH	Swiss male mice	Deathless description	Mechanism research	Maia et al. (2024)
5 mg/kg, injected on days0, 2, 4, 6, 8, 10, 12 and 14	i.p	40 mg/kg	+MA, +CH,+ HH	Swiss male mice	Deathless description	Mechanism research	Agnes et al. (2023)

**TABLE 4:** CINP model injection method of rat and mice.

Injection methods	Injection site	Configuration concentration	Dosage	Solvent	References
i.p	Abdominal cavity	1. mg/mL 2 mg/mL 4 mg/mL	2 mg/(kg·d) 2.4 mg/(kg·d) 3 mg/(kg·d) 4 mg/(kg·d) 5 mg/(kg·d) 6 mg/(kg·d) 10 mg/(kg·d)	5% glucose solution Normal saline	[3,17,36,44,71,72,74–84,87–92,94–102,105–123]
i.v	Caudal vein of the rat	1 mg/mL 2 mg/mL 5 mg/mL	2 mg/(kg·d)	5% glucose solution Normal saline	[27,28,37,51]

**FIGURE 3:**
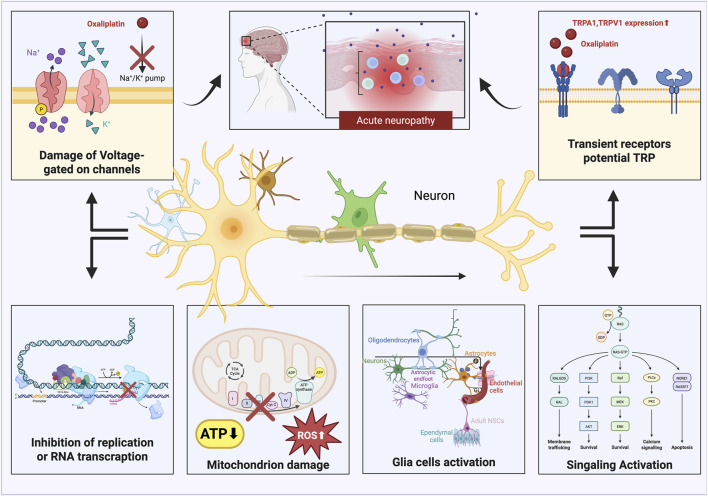
Oxaliplatin generates acute neuropathic pain by disrupting voltage-gated ion channels, activating TRP channels, reducing DNA transcription, causing mitochondrial malfunction, and leading to the emergence of reactive oxygen species (ROS). This figure was created using Bio Render.

**FIGURE 4:**
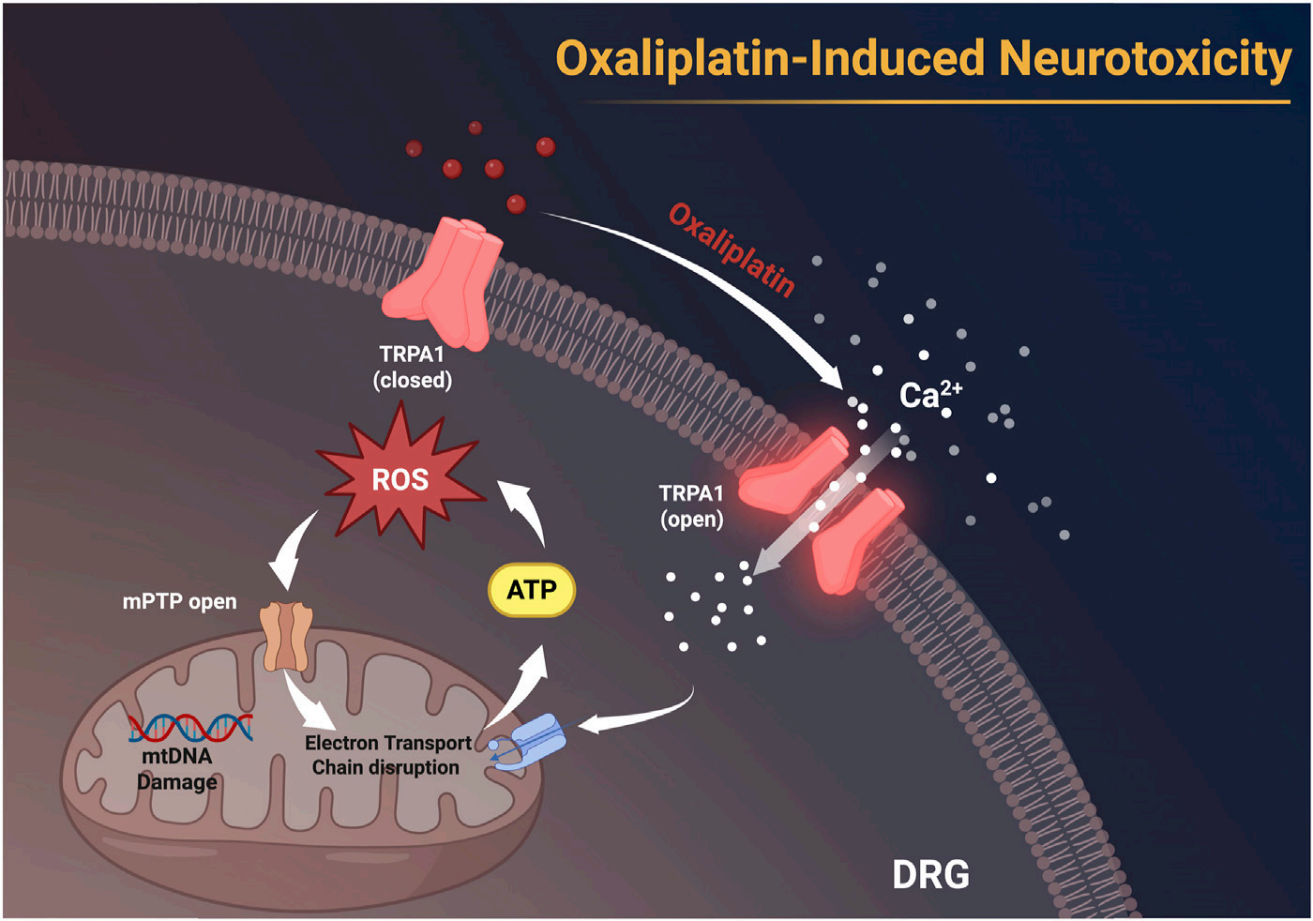
Oxaliplatin activates TRPA1 channels on the membranes of dorsal root ganglion (DRG) neurons, facilitating Ca^2+^ influx, leading to the accumulation of ROS, resulting in mitochondrial DNA (mtDNA) damage, disruption of the electron transport chain, and the opening of the mitochondrial permeability transition pore (mPTP). These alterations subsequently impede ATP synthesis and facilitate neurotoxicity. This figure was created using BioRender.

**FIGURE 5:**
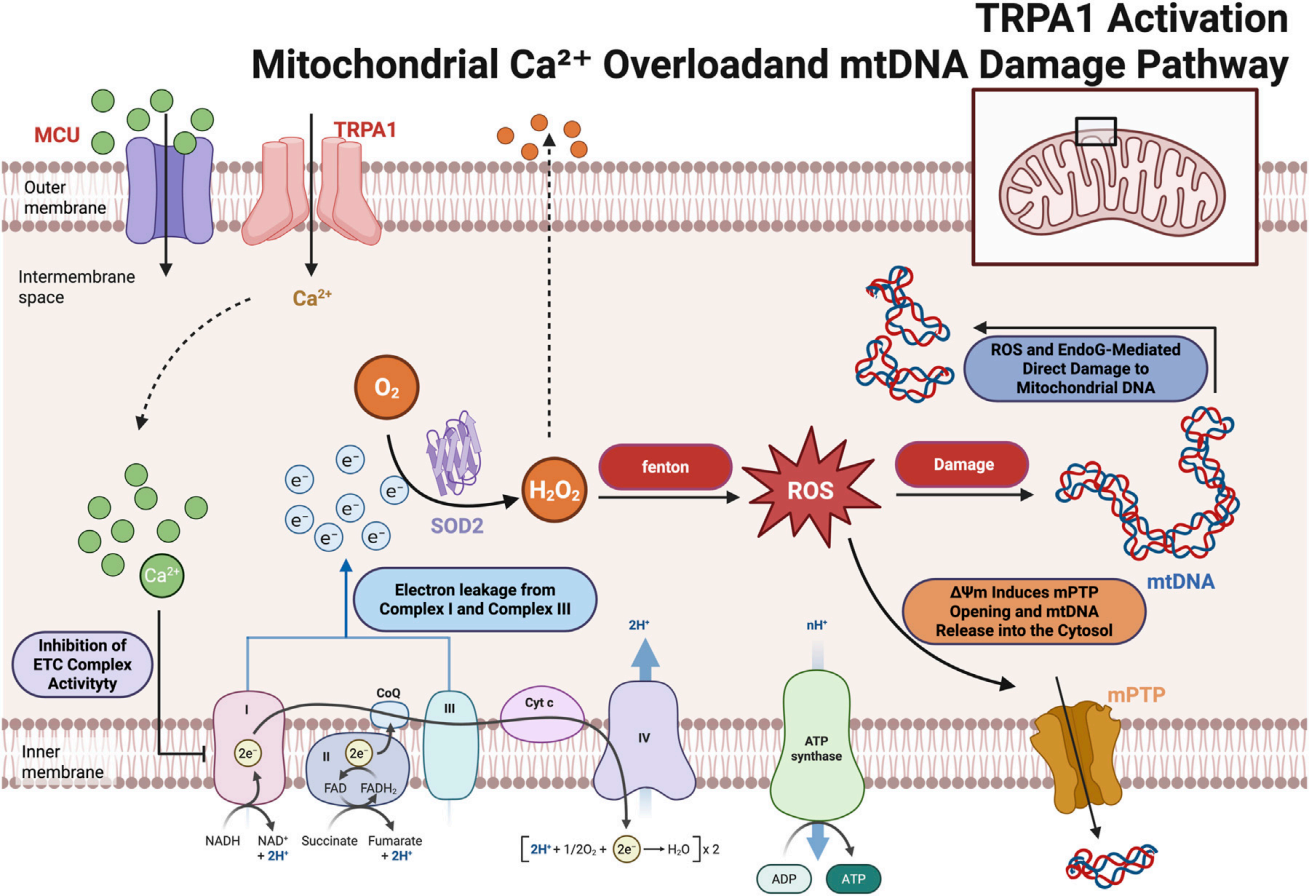
Activation of TRPA1 leads to Ca^2+^ influx, which ultimately results in mitochondrial dysfunction and neurotoxicity. mPTP:mitochondrial permeability transition pore,MCU: Mitochondrial Calcium Uniporter, mtDNA: mitochondrial DNA. This figure was created using BioRender.

OIPN can be classified into acute and chronic types, each driven by distinct mechanisms. Acute OIPN is linked to ion channel dysfunction, aberrant organic cation transporters, and glial cell abnormalities. In contrast, chronic OIPN mechanisms involve nuclear DNA damage, mitochondrial oxidative stress-induced injury, neuroinflammation through glial cell activation, and inflammation associated with gut microbiota disturbances (Yang et al., 2021). Chemotherapy-induced breakdown of the intestinal epithelial barrier results in the translocation of gut microbiota and the release of detrimental endogenous chemicals, thereby provoking the creation of pro-inflammatory mediators. This is a crucial element in the pathophysiology of CINP((Zhong et al., 2019)). Patients undergoing various chemotherapy regimens have encountered significant gut microbial dysbiosis. A notable decrease in bacteria, including Bacteroidetes, Bifidobacterium, and *Clostridium* clusters IV and XIVa, is observed (Zwielehner et al., 2011). Dorsal root ganglion (DRG) neurons express various ion channels, including voltage-gated sodium channels (Nav), potassium channels (Kv), calcium channels (Cav), chloride channels, and transient receptor potential (TRP) channels, all of which are integral to pain perception and intrinsic excitability regulation (Stevens and Stephens, 2018). Clinical studies in chronic OIPN have shown that 78% of patients present with abnormalities in Na + channels (Lee et al., 2013). The principal effects of chemotherapy-induced neurotoxicity are predominantly linked to cognitive impairment in the central nervous system in OIPN ((Cerulla Torrente et al., 2020)). The buildup of platinum-DNA adducts is regarded as a crucial element in the onset of OIPN((McWhinney et al., 2009)). Neuronal mitochondrial malfunction leading to nitro-oxidative stress is pivotal in OXA-induced neuropathy (Streckmann et al., 2018). (Figures 3–5).

Oxaliplatin treatment notably alters the expression of voltage-gated ion channels and genes involved in synaptic transmission in DRG neurons of rats (Housley et al., 2020). The activation of glial cells in the DRG, contributes to heightened inflammatory responses and increased neuronal excitability, ultimately leading to chemotherapy-induced hyperalgesia (Fumagalli et al., 2020). Oxaliplatin is believed to primarily induce apoptosis by forming DNA adducts (Yang et al., 2018). Investigations into the mechanisms underlying oxaliplatin-induced CINP focus on the pathological processes and molecular pathways in animal models, with oxidative stress playing a pivotal role in neuropathic injury.

Key indicators for evaluating and validating CINP in animal models include nociceptive abnormalities, particularly tactile allodynia thresholds assessed by electronic Von Frey testing. Neuropathic pain serves as a reliable marker for CINP severity in patients (Kerckhove et al., 2021; Selvy et al., 2021), and measuring nociceptive thresholds in live rodents offers convenience and high reproducibility (Turner et al., 2019). Selecting an appropriate animal model is thus essential for addressing research questions. Anti-inflammatory mechanisms are investigated in male Sprague-Dawley rats (on days 0, 2, and 4) (Miguel et al., 2019). Due to their simplicity in modeling, ease of behavioral assessment, and widespread use, rat and mouse CINP models have become crucial tools for studying human disease mechanisms and exploring preventive and therapeutic strategies, with significant potential for clinical applications. This work summarizes CINP models in multiple rodent species (rats and mice), detailing different dosages, pain detection techniques, and the safety of associated modeling approaches, with the objective of offering more accurate and clinically pertinent CINP models.

Animal models are commonly employed to investigate the mechanisms underlying CINP and, more importantly, to assess the efficacy of drugs in preventing or reversing CINP symptoms (Höke and Ray, 2014). While CINP has been reported to be more severe in female patients (Mizrahi et al., 2021), findings on sex differences in animal studies remain inconsistent. Some studies suggest more pronounced symptoms in males, while others report greater severity in females (Warncke et al., 2021). Therefore, future studies should incorporate both male and female animal models of CINP to improve the translational impact.

Moreover, further exploration is needed regarding factors such as chemotherapy drug type, dosage, administration route, animal model selection, and behavioral assessment methods to establish standardized experimental protocols. This will help reduce experimental bias and enable deeper investigations into current research topics, including DRG sensory neuron injury, ion channel dysfunction, and novel therapeutic approaches.

Considering the significant influence of oxaliplatin dosage on neuropathy and chemotherapeutic effectiveness, it is essential to investigate personalized precision treatment approaches further (Krishnan et al., 2005). At the typical therapeutic dosage, oxaliplatin not only precipitates neuropathy but may also result in extra adverse events. Research indicates that specific chemosensitizers can augment the therapeutic efficacy of oxaliplatin (Ge et al., 2016). Based on this, the dosage of oxaliplatin can be appropriately reduced when using sensitizers. Chemotherapy frequently induces peripheral neuropathy in cancer patients, a prevalent side effect that can severely affect their quality of life. It generally presents with symptoms including numbness, discomfort, and atypical feelings. Despite the availability of certain ways to mitigate these symptoms, CINP continues to pose a significant challenge for cancer patients. To effectively tackle this dilemma, there is an immediate necessity to create more accurate animal models. These models enable researchers to more effectively find novel therapeutic targets and clinical tactics, ultimately significantly enhancing the treatment experience for cancer patients.
